# Thermal stress and comfort assessment in urban areas using Copernicus Climate Change Service Era 5 reanalysis and collected microclimatic data

**DOI:** 10.1007/s00484-024-02639-z

**Published:** 2024-02-20

**Authors:** Tiago Silva, António Lopes, João Vasconcelos, Ata Chokhachian, Malte Wagenfeld, Daniele Santucci

**Affiliations:** 1 Centre of Geographical Studies (CEG), Institute of Geography and Spatial Planning, University of Lisbon, Rua Branca Edmée Marques, Lisbon, Portugal; 2 Associate Laboratory Terra, Coimbra, Portugal; 3 Instituto Politécnico de Leiria, Leiria, Portugal; 4 Climateflux GmbH, Munich, Germany; 5 School of Design, RMIT University, Melbourne, Australia

**Keywords:** UTCI, UHII, Heat stress, Thermal comfort, LCZ

## Abstract

In this initial study of a research project, this paper seeks to understand the thermal conditions in the cities of Lisbon and Munich, specifically focusing on Urban Heat Island intensity and on thermal comfort using the Universal Thermal Climate Index modeling data at the Local Climate Zone scale. Based on these datasets, Munich has exhibited more unfavourable thermal conditions than Lisbon. In terms of UHII, both cities have shown that low, medium, and high rise compact urban areas and bare rock or paved areas have the highest values, while sparsely built areas have the lowest. These results differ from the UTCI, which indicates that in Lisbon and Munich, these sparsely built areas as well as areas with low plants and vegetation are the most uncomfortable. In Munich, the population was exposed to very strong heat stress, while Lisbon experienced strong heat stress conditions. Conversely, low, medium, and high rise compact urban areas and densely wooded areas in Munich, and scattered trees areas and large low-rise urban areas in Lisbon, have demonstrated the lowest monthly mean and average maximum values. These results will be further explored in future studies in the city of Lisbon and cross-checked with data obtained from roving missions. This will enable a more detailed temporal and local analysis.

## Introduction

Human interaction with the thermal environment is undeniable (Jendritzky et al. 2012). This interaction primarily takes place in urban environments, where more than half of the world's population resides (The World Bank 2023). Because of climate change, urban areas, which are already susceptible to these impacts (Foshag et al. 2020), are becoming increasingly extreme hot spots (Reis et al. 2020). These extreme temperatures and induced thermal stress pose threats to human well-being and health (Foshag et al. 2020). As cities continue to grow, it is predicted that the Urban Heat Island (UHI) will intensify, thereby jeopardizing human thermal comfort (Antonini et al. 2020; Elnabawi and Hamza 2020; Huang et al. 2017; Lin et al. 2017). Oliveira et al., (2021b) found that in Lisbon the UHI is not intensified by extreme heat phenomena such as heatwaves. Therefore, understanding the thermal characteristics of urban areas is of paramount importance for promoting outdoor thermal comfort and managing outdoor heat stress (Elnabawi and Hamza 2020; Lai et al. 2020).

Since Luke Howard’s first observation of the UHI in 1833, this phenomenon has been widely studied in many cities (De Ridder et al. 2017; Deilami et al. 2018; Fahed et al. 2020; Lin et al. 2017; Lopes et al. 2013; Matzarakis and Mayer 2008; Montávez et al. 2000; Oliveira et al. 2021b, 2021a; Peng et al. 2012; Roth 2013; Santamouris 2014; Stewart and Oke 2012; Stewart and Mills 2021; Zheng et al. 2023). In Lisbon, these studies have been carried out by Alcoforado and Andrade (2008), Alcoforado and Andrade, (2006), Lopes et al. (2013) and Oliveira et al. (2021b) whereas in Munich Matzarakis and Mayer (2008) examined the urban climate of the city. Numerous studies on human outdoor thermophysiological comfort have also been undertaken around the world (Batur et al. 2022; Chokhachian et al. 2018; Colter et al. 2019; Deng et al. 2023; Emery et al. 2021; Fang et al. 2018; Geletič et al. 2018; Krüger and Rossi 2011; Lau et al. 2019a, b; Lau et al. 2022; Lau et al. 2019b; Middel et al. 2016; Oliveira and Andrade 2007; Salata et al. 2018; Sharmin et al. 2015; Zheng et al. 2023). Research in this field has led to the development of various indices over the years, such as the Universal Thermal Climate Index (UTCI), which is based on meteorological conditions and physiological criteria of the human body (COST Action 730 2012; Lai et al. 2020). In the study areas proposed here, some authors have focused on the investigation of thermal comfort, such as Oliveira and Andrade (2007), Andrade (2003), Andrade et al. (2011) in Lisbon, as well as Chokhachian et al. (2018) in Munich. These authors focused predominantly on the subjective comprehension of thermal comfort, namely thermal sensation, and thermal perception. Oliveira and Andrade (2007) found a relationship between outdoor climatic comfort, environmental and personal conditions. Andrade (2003) found an increased frequency of periods of heat discomfort in Lisbon. Chokhachian et al. (2018) found that the UTCI correlated well with thermal sensation votes, and not well with skin temperature. These authors also found that people from different origins have different expectations of thermal exposure, resulting in different thermal sensations and perception.

Cities should not be generalized concerning their thermal performance. Therefore, it is crucial to study each city at various spatial scales. According to Oke et al. (2017) and Kim and Brown (2021) these spatial scales include streets, blocks, and the neighbourhoods. The significance of employing these scales lies in their unique characteristics, which contribute differently to the climate at each level (Oke et al. 2017). More recently, another spatial scale, Local Climate Zones (LCZ), has been utilized in the study of thermal comfort and Urban Heat Island intensity (UHII) (Geletič et al. 2018; Lau et al. 2019a, b; Lau et al. 2022; Unger et al. 2018; Zheng et al. 2023). LCZ refers to a classification method for urban areas based on their climatic characteristics (Stewart and Oke 2012). This system relies on a combination of physical and land-use attributes, including building height, vegetation cover, surface materials, and water bodies. The core concept is that distinct parts of a city exhibit varying climatic properties. The use of LCZ as a spatial scale is well-suited for urban climate research (Oke et al. 2017). According to Oliveira et al., (2020) the LCZ have been used standardly as a way to represent land cover or land use classes based on their climatic properties. This is fundamental for comparison studies between urban areas around the globe since it is the equal denominator. In this paper the LCZ suitability will be tested using modelling data, as it could assist policymakers in data-deprived cities in making informed decisions regarding heat stress mitigation.

Data models can be viewed with scepticism due to intrinsic uncertainties and its simplified representation of reality (Kwok et al. 2019). Nevertheless, as exemplified by Santamouris (2014) the use of mesoscale modelling techniques is common in UHI studies because model data is easily to accessible and manageable. While some researchers may rightly argue that observational data is more precise, it must also be acknowledged that it has limitations in terms of spatial coverage. According to Kwok et al. (2019), models offer certain advantages over observational data as they maintain the inherent physical coherence among meteorological variables and allow for the simulation of various scenarios. Additionally, numerical data may provide a more consistent representation than observational data. The possibility of cross-referencing this type of data with spatial scale models, such as LCZ, enables the identification of critical areas in terms of heat stress at the city level, which is vital for local mitigation policies (Kwok et al. 2019).

In the designated study areas, the application of LCZ as a spatial scale for the investigation of UHII effects and thermal comfort has not been undertaken so far. To assess this, the decision was made to utilize data from the Copernicus Climate Change Service Era 5 Reanalysis rather than observational data. The aim is to establish an initial understanding of how these new models can accurately represent local thermal conditions and potentially identify urban morphological climatic patterns. For this purpose, the datasets UTCI and UHII were employed and analysed at the LCZ level. This approach is expected to yield innovative results and contribute to local policies for mitigating urban heat stress. Utilizing the LCZ as a spatial scale will enable policymakers to pinpoint the specific urban areas where intervention is required. The present study will focus exclusively on the Northern hemisphere summer months (June, July, and August) from 2000 to 2020. In order to validate the UTCI data field measurements were also conducted in the two cities.

To achieve the proposed goals, three objectives have been defined. I) to assess the average thermal comfort conditions in each city. II) to identify and evaluate UHII and UTCI conditions at the LCZ level. III) to contribute to other studies that seek to examine UHII and UTCI conditions using modeling data in the two cities.

The paper's structure begins with the examination of UHII by LCZ, followed by the UTCI analysis. The UTCI section will be divided into two types of analyses: a general approach to the city's average values and an LCZ-by-LCZ evaluation.

## Materials and Methods

### Study area

**Fig. 1:**
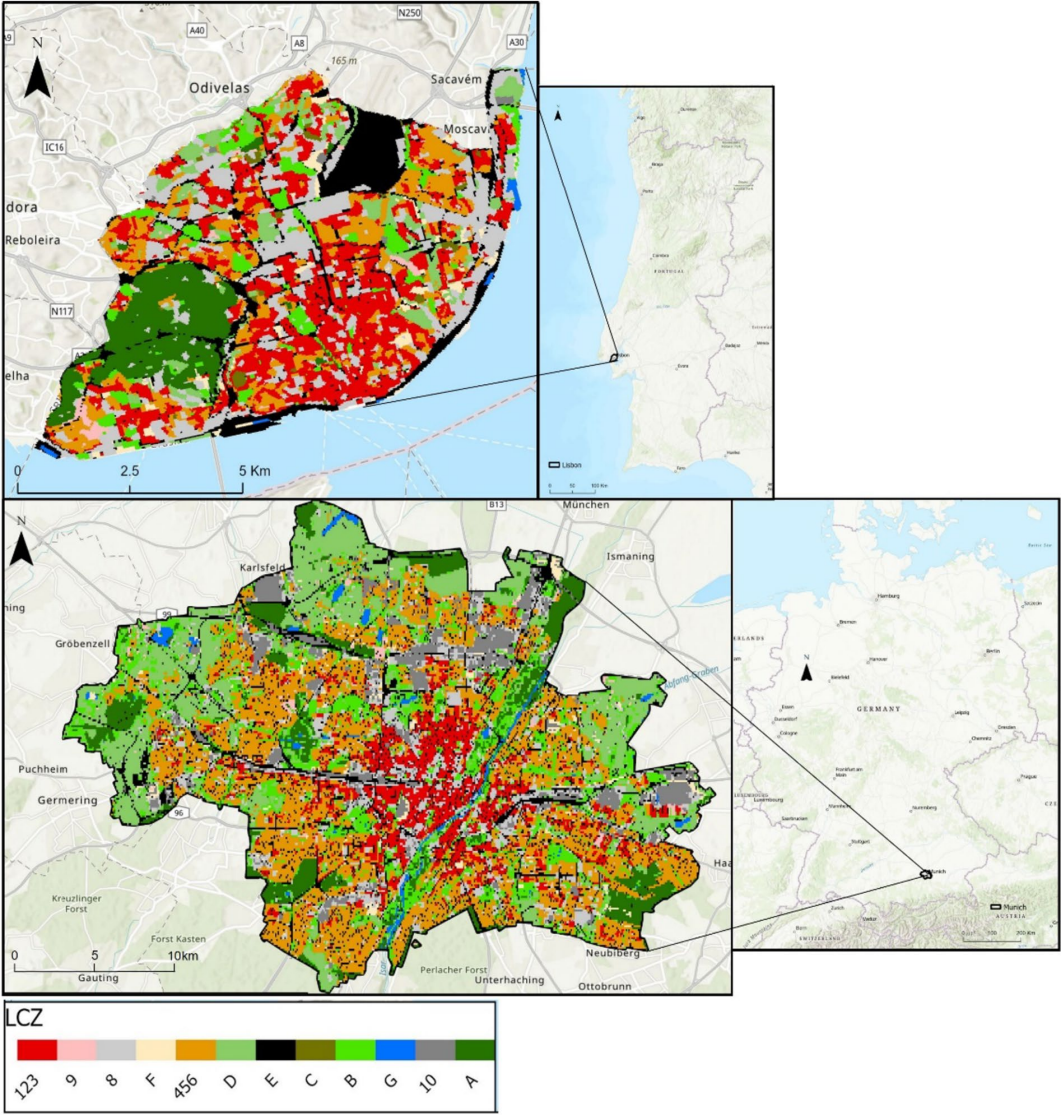
Geographical location of the study areas: **a)** Lisbon location. **b)** Munich Location. Both figures also illustrate the Local Climate Zones of each city. Lisbon LCZ Source: Oliveira et al. (2020)

**Fig. 2:**
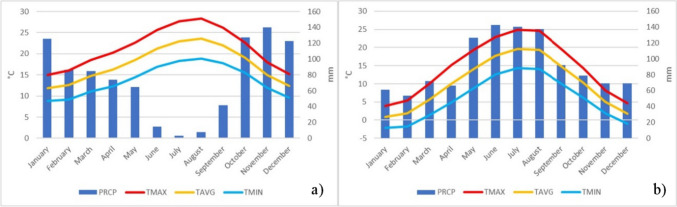
Climatic normal from the study areas. a) Climatic normal from Lisbon, 1991–2020. b) Climatic normal from Munich, 1991–2020. PRCP = Precipitation; TMAX = maximum temperature; TAVG = average temperature; TMIN = minimum temperature. Data source: NOAA

Lisbon and Munich, as previously introduced, have been selected as the study areas (Figs. 1 and 2). The comparison of cities in the research field of Urban Climatology has become increasingly necessary to generate knowledge on a regional scale regarding phenomena analysed at the local level. This is particularly interesting because, although the cities exhibit different patterns, they may share similarities that enable robust and collaborative responses. The common denominator here is the LCZ spatial scale. In the case of Lisbon, this city is situated on the western coast, while Munich is located in the centre of Europe, giving it a continental perspective. The choice of these study areas also benefits from a partnership between Zephyrus (IGOT/CEG) in Lisbon and Climateflux in Munich. This collaboration aims to investigate human thermal comfort in outdoor environments and originates from the eMOTIONAL Cities and IN-HALE projects in Lisbon and from Climate Journeys group in Munich. This partnership has led to the development of a methodology for roving missions in Lisbon. This methodology is based on the previous work of Chokhachian (2022). The studies in Lisbon will continue the tradition of micro-scale investigations into human outdoor thermophysiological comfort, building upon the work of Andrade (2003) and Oliveira and Andrade (2007).

The city of Lisbon covers an area of approximately 100 km^2^, while Munich spans 310.7 km^2^. According to the LCZ classification (Figs. 1, 2 and Table 1), both cities exhibit distinct urban characteristics. In Lisbon, a significant portion of the city consists of compact urban areas with low, medium, and high-rise buildings, covering 23.53% of the area. Another substantial segment comprises an urbanized area with large low-rise buildings, accounting for 19.86% of the city's landscape. The LCZ A, representing wooded areas, is primarily found within the Monsanto Forest. In summary, Lisbon's territory is predominantly urbanized, encompassing 72.89% of its total area. In Munich, LCZ 456 (open high, mid, and low-rise urban areas) is the most prevalent, covering 25.69% of the city. The LCZ D ranks as the second most widespread category, occupying 15.16% of the city. Munich's territory is largely urbanized, constituting 61.91% of its total area.

**Table 1:** Percentage of area of the city corresponding to LCZ in Munich and Lisbon

LCZ	Designation	Munich area %	Lisbon area %
123	Compact high, mid and low-rise urban areas	8.81	23.53
456	Open high, mid, and low-rise urban areas	25.69	14.24
8	Large low-rise urban areas	9.36	19.86
9	Sparsely built urban areas	0.75	1.14
10	Heavy industry	6.93	0.28
A	Densely wooded	8.03	10.54
B	Scattered trees	12.27	5.88
C	Bush and scrubs	-	1.02
D	Low plants	15.16	6. 74
E	Bare rock and paved	10.37	13.84
F	Bare soil or sand	1.41	2.48
G	Water	1.22	0.45

According to Beck et al. (2018) the Köppen classification in Lisbon is Csa (Hot summer Mediterranean climate) and in Munich is Dfb (Humid continental climate). In Lisbon, Fig. 2a**)** reveals that the temperature is mild in this city, with a monthly amplitude slightly exceeding 10 °C (TMAX, TAVG and TMIN). In Lisbon, August is the hottest month of the year (28.4 °C – mean maximum temperature), while January is the coldest (8.8 °C – mean minimum temperature). The annual temperature amplitude, as shown, is about 20 °C. Regarding precipitation values, Lisbon, shows a substantial monthly amplitude, about 138 mm. The summer season has very low precipitation values, with July as the driest month (2.8 mm), whereas the autumn is the wettest periods, peaking in November (140 mm). In Lisbon, the hottest period coincides with the driest season, a well-known feature of the Mediterranean climate. In turn, in Munich, Fig. 2b**)**, the temperature has a rigorous behaviour especially in the winter. The monthly amplitude is lower in the winter months (about 6 °C) and progressively higher towards the summer months (about 10 °C). In this city, July is the hottest month of the year (24.9 °C – mean maximum temperature), and January the coldest (-2.1 °C – mean minimum temperature). The annual amplitude is about 27 °C, a 7ºC higher value than in Lisbon. Precipitation-wise, Munich, shows an (about) 90 mm monthly amplitude. The summer season is the rainiest, with a notable concentration in June (142.7 mm), while the November to February period has the lowest amount of precipitation (lower value in February, 53.7 mm). Munich’s weather pattern is proper of a humid continental climate, with warm and humid conditions in the summer, and cold and in the winter.

### Data models

Due to climate change and the increasing thermal stress in urban areas the analysis of the intensity of UHI and UTCI becomes ever more important. To accomplish the outlined objectives, we conducted an analysis of the UHII and UTCI datasets.

The UHI is defined as the temperature difference between urbanized areas, typically cities, and their surrounding non-urbanized natural areas (Stewart and Mills 2021). UHII represents the amplification of elevated temperatures within urban areas, potentially posing concerns for human well-being and comfort due to heat-related risks. The UHII dataset was sourced from the Copernicus Climate Change Service and made available through Climate-ADAPT. This dataset calculates the average UHII (90th percentile) for the summer season. According to Climate-ADAPT, this dataset is derived from the subtraction of the air temperature map adjusted for height to mitigate terrain impact, from the temperature value at the 10th percentile of rural (non-water) areas, then averaged. This dataset is based on the UrbClim model by De Ridder et al. (2015) which offers a very high resolution of 100 m.

The UTCI, on the other hand, represents the equivalent air temperature in a reference outdoor setting that would trigger the same physiological responses in the human body as the current environmental conditions, incorporating factors such as air temperature, wind, radiation, and humidity (Błażejczyk et al. 2010). Notably, this index was developed within the framework of COST Action 730. The UTCI data was obtained from the Copernicus Climate Change Service and provided as a netcdf file with a 0.25-degree grid, equivalent to approximately 22 km in Lisbon's latitude and 18 km in Munich's latitude. To downscale and subset the original dataset for the study areas, we utilized the Climate Data Operator (CDO), a set of Linux command lines for manipulating and analysing climate model data. The CDO was further applied to calculate daily and monthly statistics, as detailed in subchapter 3.3.

As mentioned, these datasets were analysed at the LCZ spatial scale. Lisbon’s LCZ dataset was made accessible by Oliveira et al. (2020), while the Munich LCZ was developed using the same methodology. The LCZ model underwent validation using the random control point technique, achieving 79% precision based on 100 data points. As explained by Oliveira et al. (2020), the methodology draws on multiple datasets from Copernicus Land Monitoring Surface, such as Urban Atlas, Corine Land Cover, Dominant Leaf Type, and others. Due to limitations in the Urban Atlas classes, which do not distinguish LCZ's 8th and 10th classes, Google Maps was employed to address this issue. For simplicity and due to low heterogeneity, certain LCZ classes were aggregated. Specifically, urban classes 1, 2, 3, 4, 5, and 6 were modified to 123 and 456, representing compact urban areas and open urban areas, respectively. Additionally, in Lisbon, LCZ A, corresponding to Monsanto Forest and the airport, was excluded, with the former's cooling effect (Lopes et al. 2013) and the latter's potential exaggeration of UHII and UTCI values in mind. This model achieves a resolution of 10 m and played a pivotal role in identifying areas with the highest UHII and UTCI conditions in both cities.

### Observation data

**Fig. 3:**
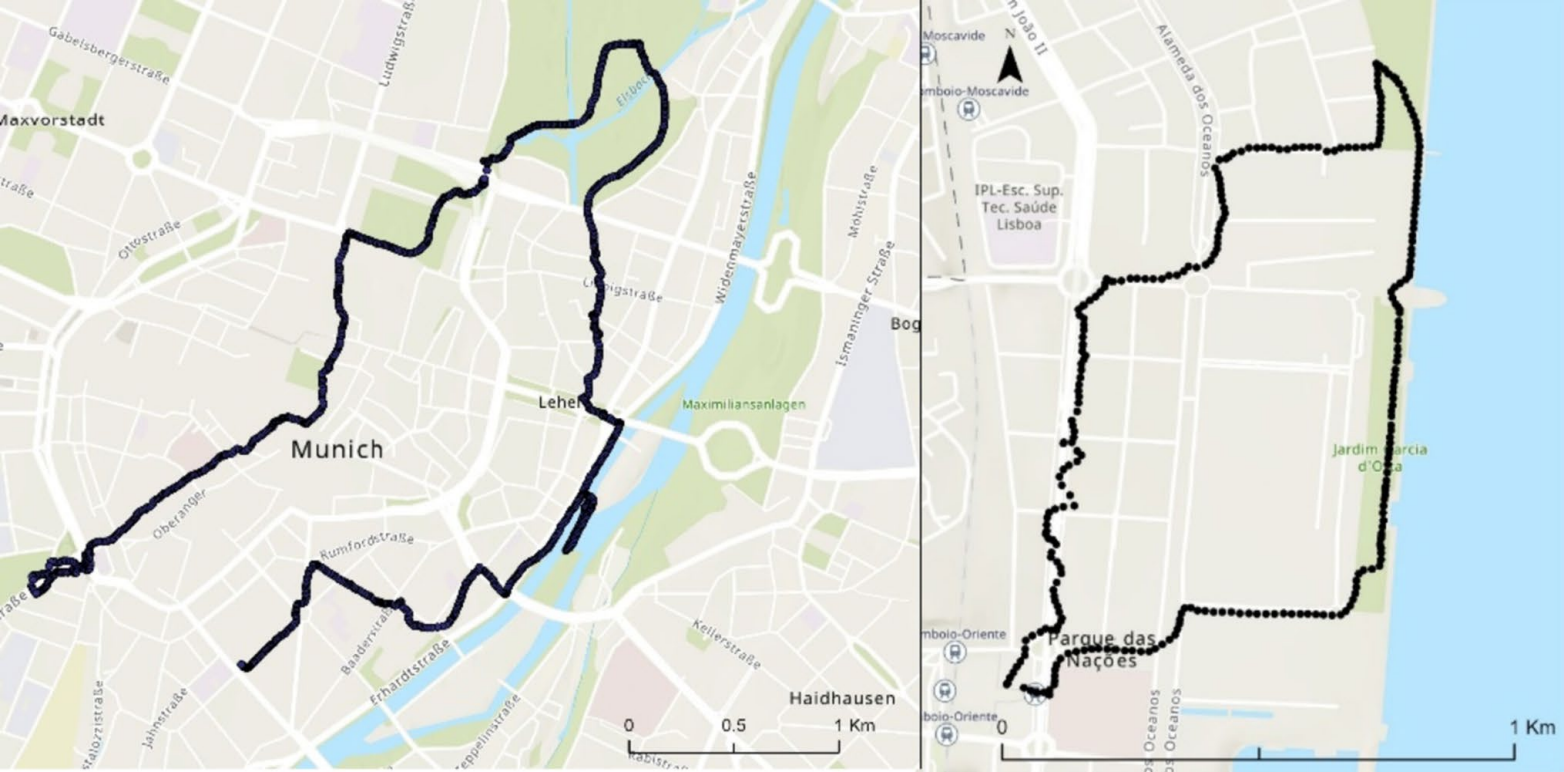
Areas where UTCI data was collected through roving missions. Left hand side: Munich. The route was run along the city centre all the way to the “Englischer Garten” and then along the Isar River. Right hand side: Lisbon. The route was run in “Parque das Nações” civil parish, through a modern urban area and then along the Tagus river

**Fig. 4:**
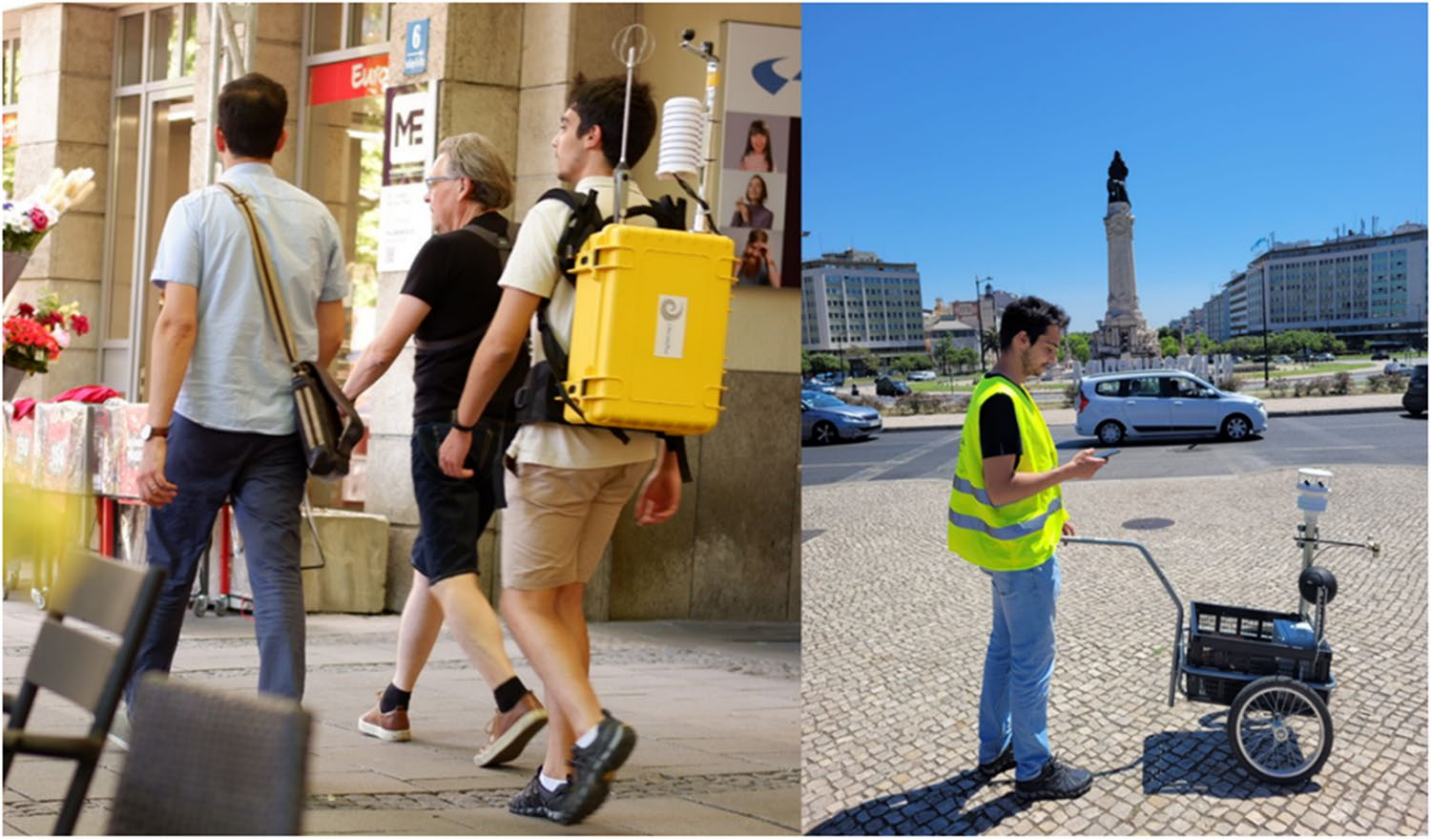
Roving missions and mobile weather stations: **a)** Munich and **b)** Lisbon

The fieldwork rationale was guided by two premises: The first to validate the Copernicus Climate Change UTCI modelling data used in this research; and the second, to prepare and test this fieldwork methodology, for more comprehensive and demanding campaigns in Lisbon. Thus, one route in each city (Fig. 3) was chosen on the basis of three premises: areas with the most diversity of LCZ as possible, proximity to the river side, and flattest areas as possible. To run the experiments, portable weather stations were used. The equipment used in Munich is the Climatewalks backpack (Fig. 4a) with microclimate monitoring sensors listed in Table 2. In Lisbon, the equipment used was a GMX500 compact weather station equipped with GPS and a CR350 datalogger, adapted to be mobile (Fig. 4b). Both weather stations enable the UTCI calculation.

**Table 2:** Equipment’s specifications used in Munich and Lisbon

Variables	Range		Resolution		Accuracy	
	Munich	Lisbon	Munich	Lisbon	Munich	Lisbon
Air temperature	-50ºC – 60ºC	-40ºC –70ºC	0.1ºC	0.1ºC	± 0.1ºC	± 0.3ºC
Wind speed	0 – 30 m/s	0.01 – 60 m/s	0.01 m/s	0.01 m/s	0.3 m/s	0.3 m/s to 40 m/s
0.5 m/s to 60 m/s						
Wind direction	0º—359º	0º—359º	1º	1º	± 5º	0.3 m/s to 40 m/s
0.5 m/s to 60 m/s						
Relative humidity	0 – 100%	0 – 100%	0.01%	1%	± 2%	± 2%
GPS	-148 dBm (aquisition), -165 dBm (tracking)	-	10 Hz	10 Hz	3 m	2.5 m
Pyranometer	0 – 2000 W m-2	300 – 3000 W m-2	0.01 W m-2	1 W m-2	1 W m-2	< 3% at 1000 W m-2
Globe thermometer	-50ºC to + 400ºC	-50ºC – 100ºC	0.01ºC	0.01ºC	± 0.7ºC	± 0.3ºC

Because fieldwork methodology is conditioned by meteorological conditions, which are determinant for thermophysiological comfort, the choice of when to carry it out had to be weighed. So, the research team faced a dilemma: either having more field days of data collection but having to spend more resources and time or doing just one run and having biased data (because it would be too influenced by the weather conditions of that day) and spending the minimum amount of time and resources. An equilibrium was able to be reached, so it was thought that three roving missions on random days (close in time) could balance the situation. Therefore, each route was run three times randomly in July to dilute possible weather effects. The measurements were taken during the hottest afternoon hours (1:30 pm to 5 pm). Although Chokhachian et al. (2018) and Stewart and Mills, (2021) recommend that roving missions not exceed much over 1 h to avoid cumulative fatigue, the decision by Chockhachian and Wagenfeld when planning the route for the Munich mission was for a longer walk of around 2 h so as to cover a larger range of urban morphologies, micro-climatic conditions and LCZ's as capturing this data was the key objective of these missions. However, the Lisbon missions did observe the 1 h timeframe', these Lisbon missions also excluded rainy or extremely hot days to preserve the instruments and the researcher’s health.

**Fig. 5:**
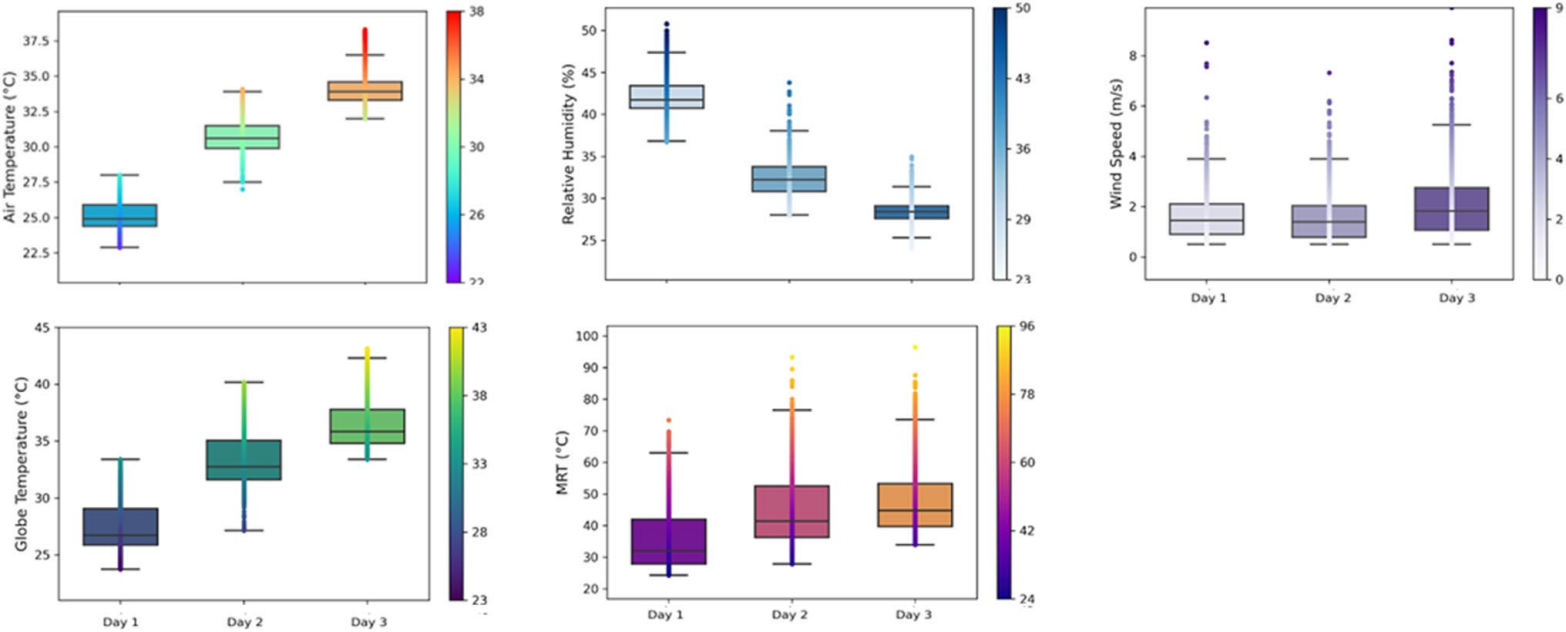
Environmental conditions during the roving missions in Munich

**Fig. 6:**
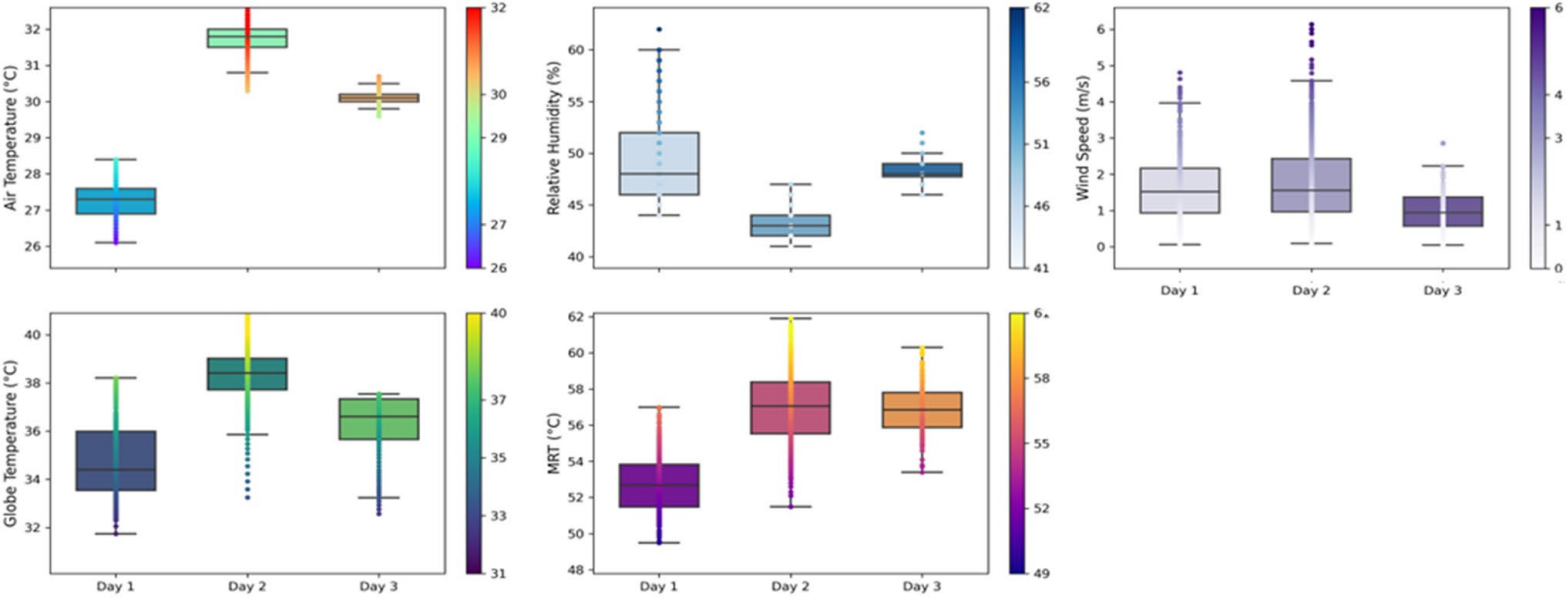
Environmental conditions during the roving missions in Lisbon

In Munich and Lisbon, the weather varied daily (Figs. 5 and 6). In Munich the air temperature had a *crescendo* and was around 25ºC (on the first day) to 35ºC (on the last day). The black Globe Thermometer and MRT had similar behaviours. The relative humidity was higher on the first day (~ 40%) and lower on the third day (~ 30%). The wind speed did not change significantly, and it was slightly higher on the last day. In Lisbon, the air temperature varied between 27ºC on the first day and 32ºC on the second. The Globe Thermometer and MRT behaved similarly. The relative humidity was higher on the first day (~ 50%) and lower on the second day (< 45%). The wind speed was lower on the last day (around 1 m/s).

This data collection method has some limitations. Regarding spatial analysis, it was not possible to cover all LCZ, and data should have been collected for the same LCZ in other areas of the city (to mitigate the effect of urban morphology). To overcome these two limitations, a wider array of roving missions to cover more areas of the city would be needed. This led to the second limitation, regarding time and resources constraints to do a more robust fieldwork. Other limitations had to do with the transportation of the equipment during the roving missions, since occasionally its stabilization could be affected by bumps or other obstacles in the pavement.

### UHII and UTCI analysis

As previously mentioned, the assessment of the UTCI (data modelling) in both study areas was conducted in several ways. Firstly, it was computed as daily means and then presented in a general context for the study areas. Secondly, as a monthly mean and thirdly, as a monthly average of the maximum UTCI values. The analysis of both datasets (UHII and UTCI) was performed using the LCZ spatial scale, which was made possible by integrating this data into Geographic Information System (GIS) software. To achieve our objectives, the Zonal Statistics Tool was employed to extract the netcdf information into the LCZ spatial scale. These indicators were instrumental in assessing the local climate conditions. LCZ G, corresponding to water bodies such as the Tagus and Isar rivers, was excluded from the statistical analysis.

## Results

### Urban Heat Island intensity

The UHII model indicates that Munich generally exhibits slightly higher values compared to Lisbon (Table 3). Munich displays a narrower range of values (1.94–2.16), thus having more uniformity, compared to Lisbon (1.70–2.34). In both cities, LCZ E demonstrates the poorest thermal performance (2.16 in Munich and 2.34 in Lisbon), while LCZ 9 consistently presents the best performance (1.94 in Munich and 1.70 in Lisbon). Notably, Lisbon showcases both the highest value (2.34) and the lowest value (1.70) between the two cities. In addition, in Munich along LCZ E, also LCZ 123 and LCZ 10 present equal values (2.16). Conversely, LCZ 9 is followed closely by LCZ A (1.96). In Lisbon, aside from LCZ E, which has the highest value, LCZ 123 ranks second with a UHII of 2.17. Importantly, these two classes exhibit a substantial range difference, expressing the poor thermal performance of LCZ E. When comparing LCZ by LCZ, it is perceivable that in Munich LCZ 456, 9, D, and F have higher values than their counterpart in Lisbon. Curiously, the worst LCZ regarding thermal performance LCZs 123 and E have higher values in Lisbon than in Munich.

**Table 3:** Urban Heat Island intensity (∆T _u-r,_ in ºC) per LCZ in Munich and Lisbon

Munich	LCZ	123	456	8	9	10	A	B	C	D	E	F
	Max	2.16	2.05	2.11	1.94	2.16	1.96	2.08	-	2.02	2.16	2.09
Lisbon	LCZ	123	456	8	9	10	A	B	C	D	E	F
	Max	2.17	1.88	2.14	1.70	-	-	1.91	1.79	1.96	2.34	2.04

### UTCI

#### Daily mean

**Fig. 7:**
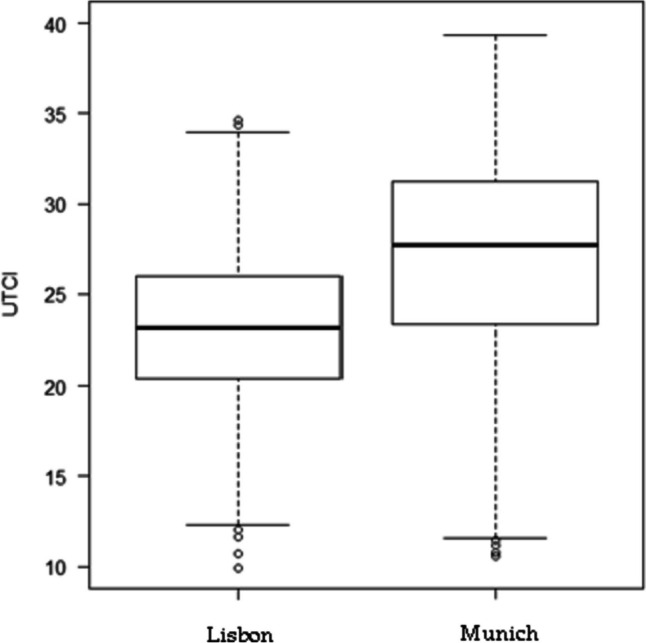
Distribution of the daily UTCI values during the summer months (June, July and August) in Lisbon and Munich between 2000 and 2020. Source: Copernicus Climate Change Service Era 5 reanalysis

The thermal comfort data illustrates notable differences between Munich (Fig. 7) and Lisbon, over the summer months from 2000 to 2020. Munich exhibits a wider range with higher maximum and minimum values compared to Lisbon. Additionally, Munich boasts a higher median value, along with elevated first and third quartiles. It's worth noting that in Munich, the first quartile surpasses even the median value in Lisbon. This underscores the uncomfortable thermal conditions prevalent in Munich. In Munich, the median corresponds to moderate heat stress (28ºC), while in Lisbon, it corresponds to conditions without thermal stress (23ºC). Decomposing the boxplot, it is also noted that in Munich 50% of the UTCI values were within 24ºC and 31ºC, emphasizing thermal stress conditions (> 26ºC – moderate heat stress). In Lisbon, 50% of the values were observed between 21ºC and 26ºC, but only a fraction reached this last value. In the upper 25% of the data, Munich reached very strong heat stress conditions (38.1—46°C) with the highest value around 39ºC. Lisbon in its worst-case scenario, reached strong heat stress conditions (32.1—38 °C) showing the highest values around 34ºC. Nevertheless, in the lower 25% of the data Munich has also encountered lower values than Lisbon (11º and 12ºC, respectively).

#### Monthly mean and monthly average of the maximum

Looking at the mean values per LCZ (Table 4) Lisbon exhibits a broader range of values between LCZ classes (16.57ºC to 17.30ºC) than Munich (17.95ºC to 18.16ºC). In Munich, LCZ 9 records the highest values (18.16 °C), closely followed by LCZ 10 and D (18.13 °C). Conversely, LCZ 123 exhibits the lowest values (17.95 °C), with LCZ A not far behind (18.01 °C). Unsurprisingly, when it comes to wooded green spaces, like LCZ A (18.01 °C) or LCZ B (18.07), they register one of the lower values. This, however, contrasts with LCZ D (18.13 °C), which is not a wooded area. In Lisbon, LCZ F and D present the highest values (17.30 °C and 17.11 °C, respectively). On the other hand, LCZ B has the lowest value (16.57 °C), followed by LCZ 8 (16.85 °C). In Lisbon, green spaces, apart from LCZ D, tend to have lower values compared to urban LCZs. Overall, in Lisbon, the UTCI is consistently lower across all LCZs when compared to Munich. In other words, urbanized areas (LCZ 123, 456, 8, 9, 10, E) in Munich have higher UTCI values compared to Lisbon’s scenario. Likewise, green areas (namely B and D) in Lisbon have lower UTCI than in Munich. Ultimately, these observations suggest that people in Lisbon tend to have better thermal comfort conditions in an outdoor environment than those in Munich, during the summer months, as reflected in this analysis.

**Table 4:** June, July and August mean UTCI between 2000 and 2020 in Munich and Lisbon. Source: Copernicus Climate Change Service (C3S)

Munich	LCZ	123	456	8	9	10	A	B	C	D	E	F
	UTCI °C	17.95	18.05	18.07	18.16	18.13	18.01	18.07	-	18.13	18.02	18.11
Lisbon	LCZ	123	456	8	9	10	A	B	C	D	E	F
	UTCI °C	17.06	16.92	16.85	17.09	17.00	-	16.57	16.99	17.11	16.93	17.30

Regarding the average values of maximum UTCI (Table 5) it is evident that both cities present values above 32 °C, signifying, as depicted in Fig. 7, that they reached strong heat stress conditions. In Munich, the thermal conditions were worst, with the highest maximum values almost reaching very strong heat stress conditions. Notably, in this analysis, Lisbon exhibits a broader range of values than Munich (33.02 to 34.11 and 36.16 to 36.55, respectively). Munich's values are also higher across all LCZs compared to Lisbon, by around 2 to 3ºC. This data suggest in Lisbon pedestrian might have better thermal comfort conditions than in Munich. In this late city, areas classified as LCZ 123 (36.16 °C) and LCZ A (36.29 °C) display the lowest values, while LCZ 9 (36.55 °C) and LCZ 10 (36.50 °C) report the highest values. In this city, urban open areas (LCZ 456) and large low rise urban areas (LCZ 8) also present lower values than sparsely wooded areas (LCZ B). In Lisbon, LCZ B (33.02 °C) and LCZ 8 (33.59 °C) record the lowest values, whereas LCZ D (34.11 °C) and LCZ 9 (34.10 °C) show the highest values. This way, it is seen that Lisbon’s LCZ B behaviour is different from what was observed in Munich.

**Table 5:** June, July and August average values of maximum UTCI between 2000 and 2020 in Munich and Lisbon. Source: Copernicus Climate Change Service (C3S)

Munich	LCZ	123	456	8	9	10	A	B	C	D	E	F
	UTCI °C	36.16	36.35	36.38	36.55	36.50	36.29	36.39	-	36.49	36.31	36.43
Lisbon	LCZ	123	456	8	9	10	A	B	C	D	E	F
	UTCI °C	34.03	33.74	33.59	34.10	33.88	-	33.02	33.88	34.11	33.76	34.03

#### Validation data

**Fig. 8:**
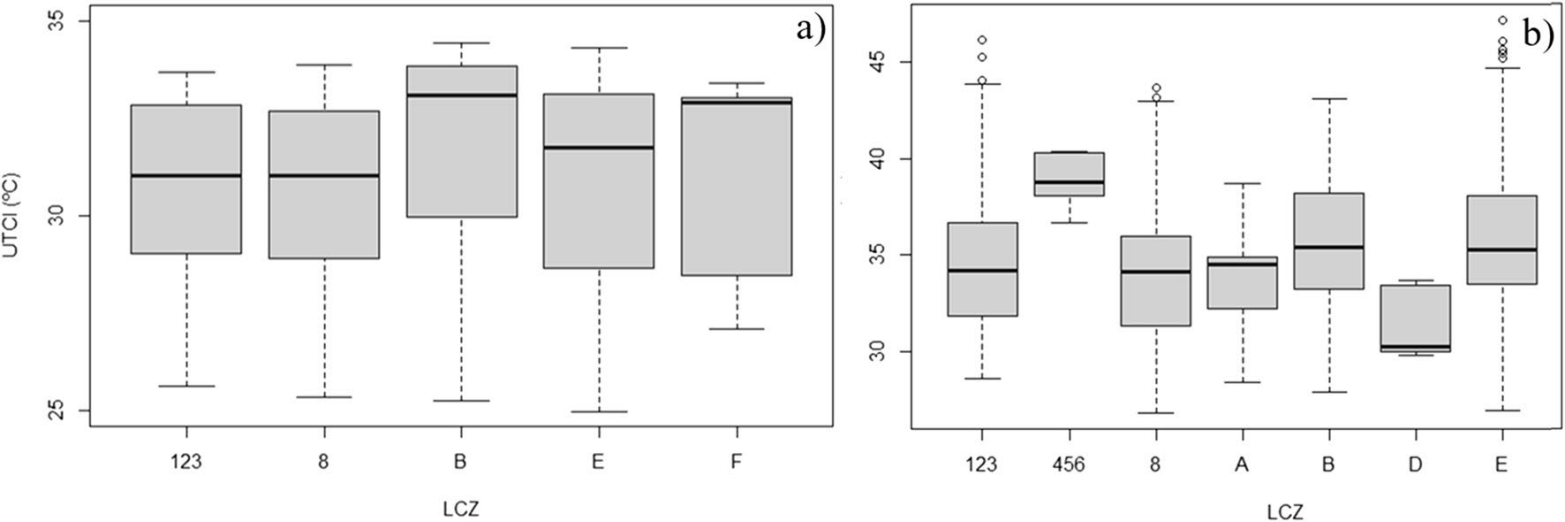
UTCI collected data in the roving missions in Lisbon (a) and Munich (b)

The observation data (Fig. 8) confirmed that Munich had higher UTCI values than Lisbon. The UTCI in Munich also had a higher amplitude of values than Lisbon. Lisbon’s and Munich’s got heat stress conditions (> 26ºC). For the same LCZs Munich had higher UTCI values than Lisbon. Almost all LCZs in Munich have median values around or above 35ºC, with much part of the data above 36ºC, stressing the very strong heat stress conditions (38.1ºC – 46ºC) that this city is able to reach. This is specially seen in LCZ 456, with data around 40ºC, followed by LCZ E and LCZ B (around 35ºC). Maximum values in LCZs 123 and E were even able to go almost as high as 45ºC. LCZ D has the lowest UTCI temperatures – median wise and has the highest value bellow 35ºC. LCZs 8 and E have the lowest minimum values shortly bellow 30ºC. In Lisbon, the median values were all above 30ºC, while the maximum UTCI values did not reach beyond 35ºC. These values, also confirm that UTCI temperatures are mostly classified as strong heat stress conditions. In Lisbon, the LCZ with the highest UTCI was LCZ B, followed by LCZ F with values around 33ºC. LCZs 123 and 8 have the lowest values (around 31ºC).

## Discussion

The thermal behaviour of Munich and Lisbon, analysed through the UHII and UTCI, reveals both disparities and a few similarities. When considering UHII, Lisbon and Munich exhibit some degree of comparability in their values. However, as explained before these models have some limitations, namely at the spatial representation, which should always be bear in mind when reading this chapter. As explained by Kwok et al. (2019), the models have inherent limitations like the simplification of reality. Albeit the city scale representation of the data, a higher resolution analysis was attempted in this paper, by using the LCZ spatial scale. Some differences between these LCZ were found that could hypothetically be explained by multiple factors despite the models’ spatial limitations, namely regarding UTCI data.

This way, in general, for both cities and models, it is evident that Munich records higher values than Lisbon, and that Lisbon exhibits a wider amplitude of values in the modelling data. Validation data agrees with the first statement and in disagreement with the second. In other words, validation data also shows that Munich records higher values, especially in UTCI, and that it has a bigger amplitude of values. In a way, the validation data shows accordance with the model data. This said, Lisbon outperforms Munich in terms of both indices, since the latter displays higher values.

A closer examination of UHII per LCZ reveals that values are consistently lower in LCZ 9 in both cities, followed by the LCZ corresponding to green spaces (LCZ C in Lisbon and LCZ A in Munich). This suggests that LCZ 9, characterized by sparsely built urban areas, offers locations where the intensity of Urban Heat Island effects could be minimized, like traditional green spaces. This may be attributed to these areas being open, featuring low-rise buildings and abundant vegetation, including trees. Within green spaces, it is possible to gauge the impact of wooded areas, notably LCZ A in Munich. In Lisbon, this class was excluded from the analysis due to its previously reported cooling effect (Lopes et al. 2013) and its location outside the urban part of the city. Conversely, both cities' LCZ E classes record the highest values, followed by LCZ 10 and 123 in Munich and LCZ 123 in Lisbon. Collectively, in both cities, areas classified as LCZ E and LCZ 123 are more exposed to the most significant UHII. This is particularly evident in LCZ 123, where most people typically live, work, or both. This heightened UHII values may be attributed to energy retention during the day, with heat not dissipating easily from these surfaces due to their highly absorbent materials and the height-to-width (H/W) ratio. Because of this, these LCZs have conditions to have higher temperatures compared to other LCZs and rural areas. As cities expand, contributing to the intensification of Urban Heat Island effects (Huang et al. 2017), it is in the most densely built urban areas where these effects are most pronounced (Antonini et al. 2020; Lin et al. 2017; Lopes et al. 2013; Zheng et al. 2023). As Zheng et al. (2023) notes, UHII conditions are closely linked to urban morphology. However, identifying areas with significant thermal anomalies does not necessarily imply that they are (un)comfortable for people. This information merely highlights parts of the city where higher temperatures prevail due to the influence of the urban environment on thermal regulation. This way, it is our belief that the city of Munich might have a more robust green infrastructure than Lisbon, which could moderate the UHII effect. Also, the wind regimes might also have some influence on the analysis at this same spatial scale.

The UTCI daily mean for the summer period appears to indicate that Lisbon can enjoy milder UTCI conditions than Munich, making it more comfortable. This is justified according to what was shown in Fig. 4 which shows that Munich experienced higher thermal stress conditions, even reaching very strong heat stress, while Lisbon encountered strong heat stress conditions. When looking at the median and quartiles, it becomes evident that all these indicators in Munich surpass those in Lisbon. For instance, Munich's median reached moderate heat stress conditions, whereas Lisbon's median indicated no thermal stress conditions. The higher UHII and UTCI values in Munich could be attributed to three conditions: first, the continental effect experienced in the summer in Munich, in contrast to Lisbon, which benefits from its proximity to the ocean, acting as a thermal regulator. In Lisbon, Andrade (2003) discussed the effects caused from this proximity in his research. This oceanic effect may also explain the differences in values within Lisbon's LCZs. Secondly, in Munich the high precipitation values in the summer, as shown in the study areas contextualization (Fig. 2), relate to high relative humidity percentages. As one of the meteorological variables used to calculate the UTCI, it’s high values may be one of the reasons why UTCI has high values in this city. Contrasting, it is seen that in Lisbon the summer period is very dry. One could argue that Lisbon should also have high humidity values due to the closeness of the ocean and the estuary, but the wind comes into play. So, the third reason is that Lisbon is very windy in the summer. This phenomenon is called the “Nortada” which stands for strong northerly winds. These winds have been largely studied by Andrade (2003), Lopes et al. (2013) and Vasconcelos (2006) and it has been found that this phenomenon strongly affects air temperature. The western most part of Lisbon is also affected by another wind regime. This second regime is characterized by westerly winds which come directly from the Atlantic Ocean and cools down that part of the city (Andrade 2003; Lopes et al. 2013; Oliveira and Andrade 2007; Vasconcelos 2006). So, even though Lisbon is known for its good weather and sunny conditions, and despite Munich being in a higher latitude than Lisbon, it has been found to have stronger thermal stress conditions during the summer. This is also observed in the study of Antonescu et al. (2021) in which is found that Munich has slightly more hours of extreme, very strong or strong thermal stress (1–1.99%) than Lisbon (0.1%-1%). These authors also show that Lisbon has the lowest the UTCI anomaly compared to other cities around Europe. However, Oliveira et al. (2022) who studied the heatwaves at a regional scale, found that Lisbon has a higher probability of having heatwaves than Munich. Nonetheless, one should be careful when such hypotheses are given since these results depend on a model with low resolution.

Despite the model’s spatial limitation, an attempt was made to increase the analysis resolution by using the LCZ. The results by LCZ show that Munich has higher UTCI values in each LCZ compared to Lisbon. Therefore, according to this model data people in Munich might have been more exposed to thermal stress conditions than in Lisbon. In the monthly mean UTCI and average maximum UTCI in Munich, LCZ 9 and 10 (plus D in the first scenario) exhibit the highest values. In Lisbon, the situation is slightly different. In the monthly mean, LCZ F and D report the highest values, whereas in the average maximum, it is D and 9 with the highest UTCI. In both cities and scenarios, there appears to be an overlap in LCZ 9 and D. These outputs could be attributed to the model resolution but could also be seen as differences in the openness of these areas, types of materials that constitute the surface and the lack of wooded vegetation. However, such hypotheses cannot be validated using this type of data. In Munich, in both scenarios, LCZ 123 and A record the lowest UTCI. This might suggest that these areas could have some characteristics that enabled to reach these values. The shade-creating effect of compact urban areas and densely wooded areas could provide that. Added to this, the wind channelling in urban areas, might decrease the UTCI values. However, observation data in these areas is required to confirm these hypotheses. In Lisbon, LCZs B and 8 exhibit the lowest UTCI in both analyses. In the city of Lisbon, green spaces, particularly LCZ B, are an effective type of green space for heat relieve (Andrade and Vieira 2007; Reis and Lopes 2019). The significance of wooded green areas in cities is also noted by Kwok et al. (2019) in their work, as they refer that during the daytime, these areas are the most favourable for outdoor thermal comfort. The validation data shows some important differences to what has been found in the modelling data. For instance, in Munich the LCZ 456 was found to have the highest UTCI, whereas LCZ D has the lowest. These results disagree with the Copernicus data. In addition, the LCZ B in this city also had high UTCI values. In Lisbon, LCZs B and F have the highest UTCI and the LCZs 123 and 8 the lowest. LCZs F and 8 showed some agreement with the modelling data. Since, the limited spatial scale and the specific urban morphological characteristics of the roved areas, a wider study in these cities using roving missions would be the ideal, despite some similarities, such as in Lisbon. The observational data has also shown that it has a serious spatial limitation and could be biased due to the urban morphological context or weather conditions. Nonetheless, they are reliable data collected directly in the field.

In research in other cities, it is worth noting that Geletič et al. (2018), Unger et al. (2018), Kwok et al. (2019) and Lau et al. (2022) have reported some different results. For example, Kwok et al. (2019) found that in Toulouse LCZ 9 and 6 are the coolest areas of the city, while LCZ 4 and 5 are the warmest. According to Kwok et al. (2019), Geletič et al. (2018) and Unger et al. (2018) found that less densely built urban areas, such as LCZ 9 and LCZs corresponding to green spaces, provided greater thermal comfort. Conversely, Geletič et al. (2018) found that the LCZ 2, 3, 5, 8 and 10 were the least comfortable. In turn, Lau et al. (2022), mentions that mid-rise LCZs, namely LCZ 2, are the most uncomfortable. They suggested that this result might be attributed to the compactness, types of materials used in these types of areas, and lack of shading. Lastly, Lau et al. (2022) also refers that LCZ D and F due to presence of vegetation and water proximity are the most comfortable LCZ in their study. Huang et al. (2017) also mentions that compact areas of the city are more uncomfortable to the population, due to reducing the wind cooling effect. This effect can also be observed in parts of the city of Lisbon, namely in Belém, as shown by Lopes et al. (2013).

The comparison of the UHII and UTCI revealed that the results of both analyses do not align much. For instance, the UHII suggests that LCZ E has the highest values in both cities, while according to the UTCI, this same LCZ reports some of the lowest values. Furthermore, LCZ 9, despite having higher UTCI values in both cities (except for the mean monthly UTCI in Lisbon), exhibits the opposite trend when analysing the UHII. The same disparity can be observed in Munich with LCZ 123. This suggests that the intra-urban temperature differences between urban and rural areas or the thermal impact of urban areas on temperature in different city areas do not necessarily correlate with (un)comfortability conditions. Instead, it indicates that individuals in these areas experience higher temperatures than they would in rural areas, potentially making them uncomfortable if no action is taken.

This difference should not impede mitigation or adaptation efforts, as these models should be considered complementary. Both should be carefully analysed and always used in conjunction with spatial scales as a backdrop. Therefore, considering the divergent literature results compared to the findings in this paper, further studies are necessary, especially considering observational data obtained, for example, through roving missions. Roving missions have already commenced in Munich by Chokhachian et al. (2018) and Santucci and Chokhachian (2019) while in Lisbon, they have been conducted as part of this project. The results of these missions will be published in the subsequent phases of this research. Kwok et al. (2019) argues that point measurements, such as those conducted in Lisbon, do not capture spatial variations, providing yet another reason to adopt this methodology in this city.

## Conclusion

Modelling data are a useful tool to evaluate climate data as well as thermal comfort conditions. However, they may present some limitations, like having low spatial resolution. This was particularly notorious in the UTCI dataset which has a low resolution to be analysed at the city scale. Nonetheless, an attempt was made to identify at the city scale, areas where the thermal comfort is generally lower or higher, which can possibly be the target of heat mitigation efforts. This way, it has been found that the UTCI and UHII values are higher in Munich than in Lisbon, which in turn has higher amplitudes. Based on the findings, it appears that Munich experiences more adverse thermal and comfort conditions than Lisbon. Munich was impacted by very strong heat stress conditions, a category higher than that observed in Lisbon. The difference between the cities might be explained by three factors: the continentality of Munich compared to the proximity to the ocean in Lisbon, the high relative humidity values in Munich and lower in Lisbon, and the strong wind effects in Lisbon.

The intracity analysis revealed that areas classified as LCZ 9 have the lowest UHII in both cities, while LCZ E have the highest. In terms of thermal comfort, it has been found that according to the monthly mean and average maximum UTCI per LCZ, it is evident that in Munich, LCZ A and 123 report the lowest values, while in Lisbon it is LCZ 8 and LCZ B. Conversely, in both cities, LCZ 9 and D exhibit the areas with the highest values. These outcomes contrast with prior research studies conducted in other cities, underscoring the need for further investigations utilising alternative methodologies such as roving missions, able to collect data. One limitation of the observational data is that the data collection is spatially very conditioned. This way, more observation data is needed to confirm these hypotheses, and to cover more areas of the studied cities. Also, additional data could also enable a seasonal analysis of thermal comfort instead of a summer only study.

The use of LCZ as a spatial scale when studying UHII and UTCI can assist city planners in comprehending local urban thermal characteristics and identifying potential areas for mitigating heat stress. This will be explored further in future papers.

## Data Availability

Part of data that support the findings of this study are available from Climateflux. Restrictions apply to the availability of these data, which were used under license for this study.
